# Mechanical and Electrical Characteristics of Lightweight Aggregate Concrete Reinforced with Steel Fibers

**DOI:** 10.3390/ma14216505

**Published:** 2021-10-29

**Authors:** Se-Hee Hong, Jin-Seok Choi, Tian-Feng Yuan, Young-Soo Yoon

**Affiliations:** School of Civil, Environmental and Architectural Engineering, Korea University, 145 Anam-ro, Seongbuk-gu, Seoul 02841, Korea; bestshhong@korea.ac.kr (S.-H.H.); radiance@korea.ac.kr (J.-S.C.)

**Keywords:** lightweight aggregate, steel fiber, equilibrium density, dry density, compressive strength, flexural strength, electrical conductivity, shielding effectiveness

## Abstract

There is increased interest in applying electromagnetic (EM) shielding to prevent EM interference, which destroys electronic circuits. The EM shielding’s performance is closely related to the electrical conductivity and can be improved by incorporating conductive materials. The weight of a structure can be reduced by incorporating lightweight aggregates and replacing the steel rebars with CFRP rebars. In this study, the effects of lightweight coarse aggregate and CFRP rebars on the mechanical and electrical characteristics of concrete were investigated, considering the steel fibers’ incorporation. The lightweight coarse aggregates decreased the density and strength of concrete and increased the electrical conductivity of the concrete, owing to its metallic contents. The steel fibers further increased the electrical conductivity of the lightweight aggregate concrete. These components improved the EM shielding performance, and the steel fibers showed the best performance by increasing shielding effectiveness by at least 23 dB. The CFRP rebars behaved similarly to steel rebars because of their carbon fiber content. When no steel fiber was mixed, the shielding effectiveness increased by approximately 2.8 times with reduced spacing of CFRP rebars. This study demonstrates that lightweight aggregate concrete reinforced with steel fibers exhibits superior mechanical and electrical characteristics for concrete and construction industries.

## 1. Introduction

With the development of information and communication technology, the use of electronic devices and interest in electromagnetic interference (EMI) shielding have increased in recent years. EMI causes electromagnetic (EM) waves to explode an electric circuit, resulting in its destruction. When EM waves are generated from outside a building and penetrate it, electronic devices inside the building may malfunction or break down because of EMI [1,2]. EMI shielding is applied to various fields, such as medical equipment, communication, and military facilities, to prevent this phenomenon [1,3,4]. However, most buildings are constructed with reinforced concrete, and therefore, it is necessary to evaluate the EMI shielding performance of concrete [5].

Three phenomena occur when an EM wave is incident on concrete: absorption, reflection, and multiple reflections. Absorption is caused by conduction, dielectric, and magnetic losses. EM waves react with the electric charge when they are incident on concrete, and the microcurrent flows instantaneously. This reaction induces electrical resistance loss, and the concrete eventually absorbs the EM waves [4,6,7,8]. Some researchers [9,10,11] reported that the better the electrical conductivity of concrete, the better the shielding effectiveness. Therefore, it is essential to make concrete electrically conductive to improve its EMI shielding performance.

Ordinary concrete has high electrical resistance and low electrical conductivity, and the electrical conductivity of concrete can be improved by incorporating conductive materials. Conductive materials range from the macroscale to the nanoscale, and conductive fibers include carbon nanotubes (CNTs), carbon fibers, and steel fibers [12]. Jung et al. [9] experimentally evaluated the electrical conductivity and shielding effectiveness of ultrahigh performance concrete (UHPC) by incorporating CNTs. In addition, they applied the electromagnetic compatibility theory and compared the test results with the numerical results. The percolation threshold of CNTs ranged between 0.8 and 1.0 wt.%, and the shielding effectiveness significantly improved as the CNT content increased within the percolation threshold. Yoo et al. [10] evaluated the shielding effectiveness of high-performance fiber-reinforced cement composites by varying the carbon fiber content. The 0.1% carbon fiber exhibited no significant effect, but the 0.3% carbon fiber caused an approximately 15% improvement in shielding effectiveness at 1.0 GHz. Choi et al. [3] assessed the effect of steel fibers on the shielding effectiveness of concrete specimens by incorporating 0.75% and 1.50% steel fibers. The steel fibers increased the shielding effectiveness at high frequencies, and their effects became more significant with an increase in the steel fiber content. Additionally, an experimental evaluation was performed by setting the concrete thickness and reinforcement diameter as factors influencing the shielding effectiveness. Zhang and Sun [13] evaluated the effect of the steel and carbon fibers on cementitious composites between the 0.08 and 10.0 GHz frequency bands. They observed that, when 1% and 3% steel fiber contents were used, the shielding effectiveness increased by 10 and 50 dB, respectively. Within 1% carbon fiber, the shielding effectiveness improved as the carbon fiber content and frequency increased. Ozturk et al. [14] used an electric arc furnace slag, a type of steel slag, as a fine aggregate to assess its effect on the shielding effectiveness of mortar. They found that the electric arc furnace slag improved the shielding characteristics, owing to the high iron content in the electric arc furnace slag.

The interest in lightweight characteristics for structures is growing of late in the concrete and construction industries. A representative method for reducing the weight of structures is to utilize lightweight aggregates, and many studies on lightweight aggregate concrete have been conducted [15,16,17,18,19,20,21]. However, lightweight aggregates are not only vulnerable to external forces because of the numerous pores distributed inside them but they also absorb mixing water from concrete, reducing the concrete’s fluidity [16,22,23,24,25,26]. Consequently, the porous nature of lightweight aggregates reduces the concrete’s strength, owing to degradation in quality. Therefore, the water-absorbing characteristics of lightweight aggregates must be controlled during concrete mixing to secure high-quality concrete. Another way to achieve low structural weight is to replace the steel rebars with carbon-fiber-reinforced polymer (CFRP) rebars. CFRP rebar is composed of a carbon fiber and resin matrix, and its weight is approximately 20–25% of steel rebar’s weight [27]. Recently, CFRP rebars have been in the spotlight in the construction industry because of their lightweight, superior corrosion resistance, and high tensile strength [27,28,29,30]. However, most researchers evaluated the bond capacity and structural performance of concrete reinforced with CFRP rebars, but studies on EM shielding have not been conducted extensively.

In this study, the research items largely focused on the density and mechanical and electrical characteristics of concrete. First, the variation in the density of concrete according to the incorporation of lightweight coarse aggregates was analyzed. Next, steel fibers were added to lightweight aggregate concrete, which is vulnerable to external forces, to evaluate the strength characteristics. Subsequently, the effects of the lightweight coarse aggregate and steel fiber on the electrical resistivity and shielding effectiveness were evaluated in terms of electrical characteristics. Furthermore, the effects of the reinforcement type and spacing on shielding effectiveness were analyzed.

## 2. Experimental Program

### 2.1. Mix Design and Materials

**Figure 1:**
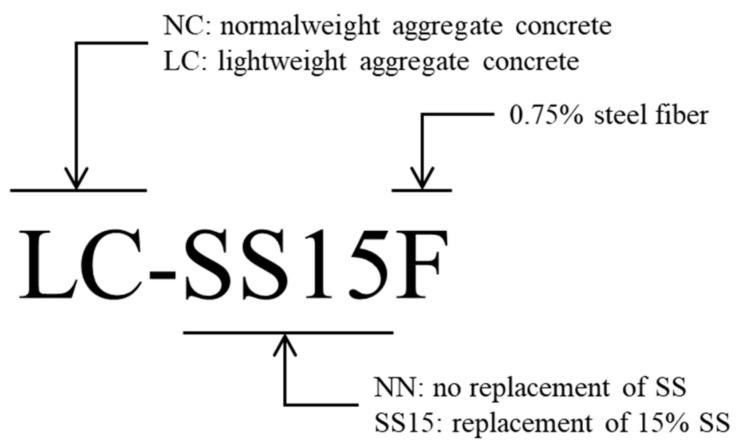
Designation of test specimens.

The mix proportions of the concrete used in this study are listed in Table 1. The water-to-binder ratios were 0.325 and 0.200, and steel slag powder was used as the binder. Several studies used steel slag powder to assess the microstructure [31,32], strength [33,34,35], and durability [32] of concrete based on the replacement ratio; almost no degradation in strength and durability occurred when the replacement ratio of steel slag was within 15%. Based on these findings, concrete mixes were designed in this study by applying the replacement ratio of steel slag at 15% [31,32,33,34,35]. Additionally, variables were set by substituting the coarse aggregate with bottom-ash-based lightweight coarse aggregates to reduce the weight of concrete containing steel slag. Furthermore, 0.75% hooked-end steel fibers were mixed to improve the ductile performance of the concrete, and tests on a total of five concrete groups of mixes were performed. Figure 1 shows the specimen identification set in this study. The reference concrete mix, in which the replacement ratio of steel slag was 0%, is denoted NN, and the specimen produced with 15% steel slag is represented by SS. F is placed at the end for specimens containing steel fibers. NC and LC are marked at the first part for specimens containing coarse aggregate and lightweight coarse aggregate, respectively.

**Figure 2:**
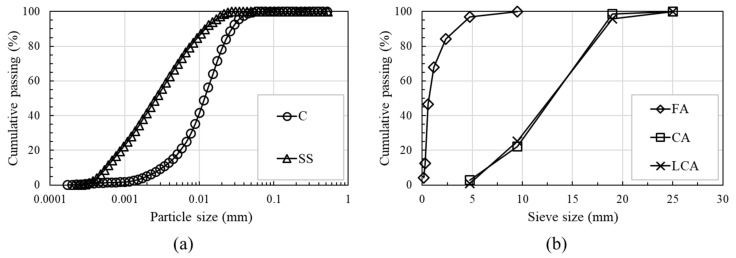
Particle size distributions of binders and aggregates: (**a**) binders; (**b**) aggregates.

Figure 2 shows the particle size distributions (PSDs) of the binders (cement and steel slag) and aggregates (fine, coarse, and lightweight coarse aggregates) used in this study, and Table 2 lists the chemical and physical properties of the binders and aggregates. The cement was Type I ordinary Portland cement with a Blaine of 3413 cm^2^/g. The steel slag powder had a Blaine of approximately 4893 cm^2^/g, and it was obtained by pulverizing steel slag aggregate using ball mill equipment (Chemius Korea Co. Ltd., Gurye, Korea) [32,34]. River sand was used as the fine aggregate. The PSDs of the aggregates satisfied the upper and lower limits recommended in ASTM C33 [36] and ASTM C330 [37]. The fineness moduli of fine, coarse, and lightweight coarse aggregates were 2.9, 6.6, and 6.7, respectively. Hooked-end steel fibers with a diameter of 0.55 mm, aspect ratio of 64, and tensile strength of 1250 MPa were used. The target slump of all concrete mixes was set to 180 ± 30 mm. A polycarboxylate-based superplasticizer with a density of 1.07 g/cm^3^ and an air-entraining agent were used. In contrast, lightweight aggregates exhibit a significant moisture-absorbing property and have many pores inside. This property results in fluidity loss during concrete mixing, and it increases the amount of superplasticizer added to the mixture [16,38]. However, in this study, the mixture was prepared according to the method for manufacturing lightweight aggregate concrete developed by Hong et al. [15] to minimize fluidity loss and improve concrete quality. The process of manufacturing lightweight aggregate concrete comprises three stages: (1) performing dry mixing of all aggregates for uniform dispersion, (2) mixing half of the water to let it penetrate into the lightweight coarse aggregates, and (3) mixing the remaining water with the superplasticizer and all binders. Table 3 lists the details of the reinforcements used in this study.

### 2.2. Test Methods and Preparation of Specimens

In this study, three categories (density, mechanical properties, and electrical properties) were applied to evaluate the effects of steel slag, lightweight coarse aggregate, and steel fiber on concrete. The density of the concrete was determined according to ASTM C567 [39]. The molds were removed 24 h after concrete casting to evaluate the oven-dry density of the concrete specimens. Next, the mass of each cylinder specimen in water was measured, and the water on the surface of the specimen was removed to determine the saturated-surface-dry (SSD) mass of the specimen. The specimen was then dried at 110 ± 5 °C for three days using oven (DAIHAN SCIENTIFIC Co. Ltd., Wonju, Korea). Three oven-dry density test specimens were prepared, and the average value was computed. Equation (1) was used to calculate the oven-dry density.

(1) $$O_{m}=\left(D\times 997\right)/\left(F-G\right)$$

where $O_{m}$ is the oven-dry density, D is the mass of the oven-dried cylinder after four days after casting, F is the mass of the SSD cylinder one day after casting, and G is the apparent mass of the suspended-immersed cylinder one day after casting.

Cylinder test specimens were prepared and cured under standard conditions for seven days to evaluate the equilibrium density. Next, each specimen was immersed in water at 23 ± 2 °C for one day. The specimen was then exposed to 23 ± 2 °C and a relative humidity of 50% ± 5% for 28 days after casting. Similar to oven-dry density, the equilibrium density was averaged using three specimens. The equilibrium density was calculated using Equation (2).

(2) $$E_{m}=\left(A\times 997\right)/\left(B-C\right)$$

where $E_{m}$ is the equilibrium density, A is the mass of the cylinder as dried at 28 days after casting, B is the mass of the SSD cylinder at 7 days after casting, and C is the apparent mass of the suspended-immersed cylinder at 7 days after casting.

The compressive strength of the concrete was determined according to ASTM C39 [40]. A universal testing machine (UTM/MTS, Minneapolis, MN, USA) with a maximum capacity of 200 t was used as the experimental equipment, and the loading rate was set to 0.35 mm/min. Six cylinders, each with a diameter of 100 mm and a height of 200 mm, were manufactured for each concrete mix. The 7- and 28-day compressive strengths of the three cylinders were measured. The flexural strength of the concrete was evaluated according to ASTM C1609 [41]. The loading equipment was the same UTM used for the compressive strength tests, and the loading rate was set to 0.20 mm/min. Each specimen was prismatic with a length of 400 mm and a cross-sectional area of 100 mm × 100 mm, and the flexural strength was determined at 28 days. In addition, the deflection of each test specimen was measured using a linear variable differential transformer. The load was applied after the peak load until the deflection of the specimen reached 2 mm. The toughness of the concrete reinforced with steel fibers was then determined from the area under the load–deflection curve.

**Figure 3:**
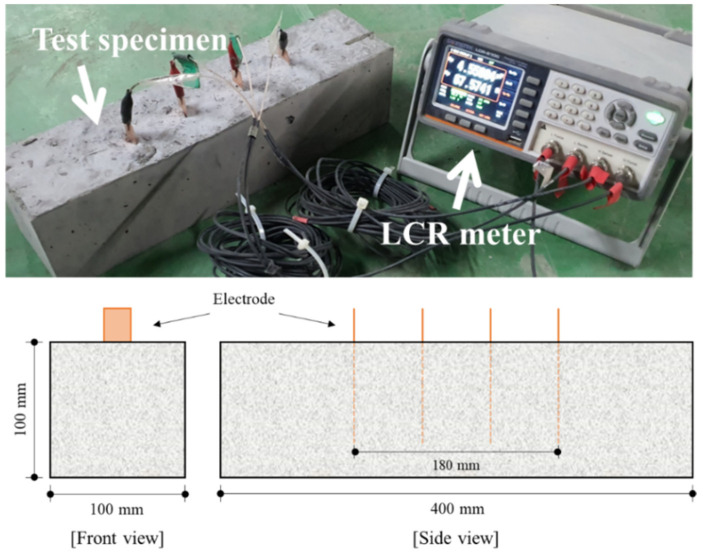
Measurement of electrical resistance of concrete.

The electrical conductivity and shielding effectiveness were evaluated for the electrical properties of concrete. Figure 3 depicts the measurement of the electrical resistance of concrete. Each prismatic concrete specimen had a volume of 100 mm × 100 mm × 400 mm, and four copper plates, each with an area of 20 mm × 100 mm, were embedded in the specimen in spacings of 60 mm [42,43]. The four-probe method was used to determine the electrical resistance of concrete, and alternating current in the 100 kHz band was applied to control the polarization effect [34]. The electrical resistance was measured using an inductance–capacitance–resistance (LCR) meter (GWINSTEK, Taiwan, China). The electrical resistivity was calculated based on Equation (3) using the electrical resistance determined at the designated age. The electrical conductivity was derived as expressed by Equation (4) by adopting the inverse of the electrical resistivity.

(3) ρ=RA/l

(4) σ=1/ρ

In Equations (3) and (4), ρ is the electrical resistivity of concrete, R is the electrical resistance of concrete recorded by the LCR meter, A is the contact area of the concrete with the embedded copper plate, l is the spacing interval between the copper plates, and σ is the electrical conductivity.

**Figure 4:**
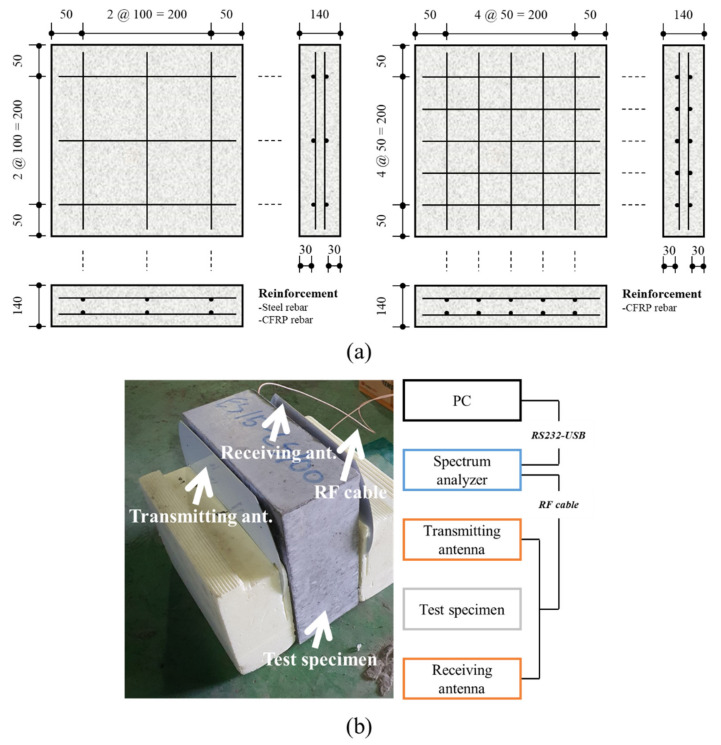
Details for measuring shielding effectiveness of concrete: (**a**) specimen details; (**b**) test setup.

A panel with a cross-section of 300 mm × 300 mm and a thickness of 140 mm was manufactured to simulate the wall structure [3]. Figure 4a shows the details of the specimens. Steel and CFRP rebars with a diameter of 10 mm were used as reinforcement. By setting the concrete cover of the steel and CFRP rebars to 30 mm, the concrete cover values satisfied the recommendations of ACI 318-19 [44] and ACI 440.1R [45], respectively. In addition, all the specimens were reinforced in two layers by arranging both rebars at 100 mm intervals, satisfying the maximum, minimum, and clear spacings and the reinforcement ratio. Furthermore, specimens containing CFRP rebars at 50 mm spacings were manufactured to evaluate the variation in shielding effectiveness with the reinforcement interval. The test specimens used for assessing the shielding effectiveness were cured for 28 days at 20 ± 1 °C and a relative humidity of 60% ± 5% and dried in an oven at 60 °C for 3 days to remove the moisture [3,9]. The error of the experimental result values at the laboratory level was minimized using the log spiral antenna and measurement software that could measure 20–80 dBm as reported by Choi et al. [3]. In addition, the shielding effectiveness was measured immediately before the experiment as a reference without a test object, and calibration was performed [10]. Additionally, as shown in Figure 4b, by matching the center of the antenna with the center of the test specimen, measurement errors induced by the dynamic range characteristics of the antenna were controlled [3,10]. In this study, measurements were performed at 0.1 GHz intervals between 0.4 and 1.4 GHz [3]. The measurement results were expressed as the shielding effectiveness in the software, and the value was derived using Equation (5).

(5) $$SE=-10\log\left(P_{t}/P_{i}\right)$$

where SE is the shielding effectiveness, $P_{t}$ is the transmitted power, and $P_{i}$ is the incident power.

## 3. Experimental Results and Discussion

### 3.1. Density Characterisitcs

#### 3.1.1. Equilibrium Density

**Figure 5:**
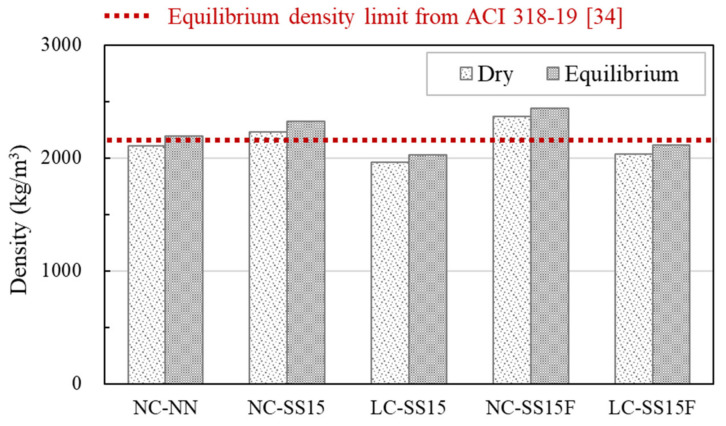
Test results of concrete density.

Figure 5 shows the equilibrium densities of all the concrete specimens. The reference specimen, NC-NN, had an equilibrium density of 2197 kg/m^3^, and the equilibrium density increased to 2323 kg/m^3^ when steel slag was substituted by 15%. LC-SS15 had the lowest equilibrium density of 2029 kg/m^3^. If the amount of the unit binder is increased, the concrete weight can be reduced using bottom-ash-based lightweight coarse aggregate, whose density is approximately 30% lower than that of conventional coarse aggregate [15]. When steel fibers were added, NC-SS15F showed an increase of approximately 4.9% compared to NC-SS15, and LC-SS15F showed an increase of approximately 4.5% compared to LC-SS15. In particular, LC-SS15F had an equilibrium density of 2119 kg/m^3^, which was approximately 3.6% lower than that of NC-NN despite the increase in density caused by the steel fibers. ACI 318-19 [44] limits the maximum equilibrium density of lightweight aggregate concrete to 2160 kg/m^3^. In this study, the equilibrium densities of LC-SS15 and LC-SS15F, which were mixed by 100% substitution of coarse aggregate with lightweight coarse aggregate, were 2029 and 2119 kg/m^3^, respectively. These lightweight aggregate concrete specimens satisfied the limit stated above.

#### 3.1.2. Dry Density

The dry density showed similar trends to the equilibrium density, but the dry density values were generally lower than the equilibrium density values for all concrete mixes. The dry densities of NC-NN, NC-SS15, and NC-SS15F decreased by 93, 90, and 72 kg/m^3^, respectively, compared to the equilibrium density. In addition, the dry densities of LC-SS15 and LC-SS15F mixed with lightweight coarse aggregate decreased by 62 and 84 kg/m^3^, respectively. LC-SS15 showed a decrease in dry density by approximately 12% compared to that of NC-SS15. Kim et al. [18] also observed that the dry density was decreased approximately 16% when replacing with lightweight aggregates. Through the use of lightweight aggregates, it is possible to expect to reduce the weight of concrete.

### 3.2. Mechanical Characterisitcs

#### 3.2.1. Compressive Strength

**Figure 6:**
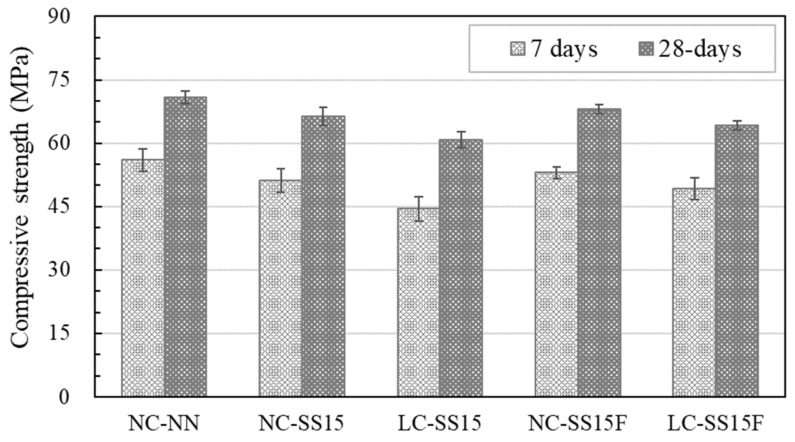
Compressive strength values at different curing ages.

Figure 6 shows the compressive strength test results for all the concrete specimens. At the initial age (seven days), the NC-NN developed a compressive strength of approximately 56 MPa. However, when the cement was partially substituted with steel slag, a decrease in strength was observed, and the compressive strength of NC-SS15 decreased by approximately 8.6% compared to that of NC-NN. This strength reduction was attributed to the low hydraulicity of the steel slag rather than the cement [31,35]. When steel slag was added, the amount of cement used was reduced, delaying the hydration reaction and limiting portlandite (Ca(OH)_2_) production [35,46]. Many researchers [31,32,33,34,35] have reported that incorporating steel slag delays the hydration reaction of concrete based on the X-ray diffraction (XRD), mercury intrusion porosimetry (MIP) analyses, and penetration resistance tests, which determine the initial and final sets.

LC-SS15, considered for weight reduction of concrete, exhibited the lowest compressive strength despite the low water-to-binder ratio. This is because lightweight coarse aggregates have a lower density than coarse aggregates, contain numerous pores, and exhibit low strength resistance [16,47]. Hong et al. [15] reported that the compressive strength decreased by approximately 7% or higher when the coarse aggregate was wholly replaced with a lightweight coarse aggregate, and Kim et al. [16] observed that the compressive strength decreased by 21%. The steel fibers appeared to improve the compressive strength. At all ages, the compression resistance of NC-SS15F and LC-SS15F improved. At 28 days, the compressive strength of NC-SS15F increased by approximately 2.7% compared to that of NC-SS15, and the compressive strength of LC-SS15 increased by approximately 5.5% compared to that of LC-SS15. LC-SS15F exhibited compressive strength characteristics similar to those of NC-SS15.

#### 3.2.2. Flexural Strength

**Figure 7:**
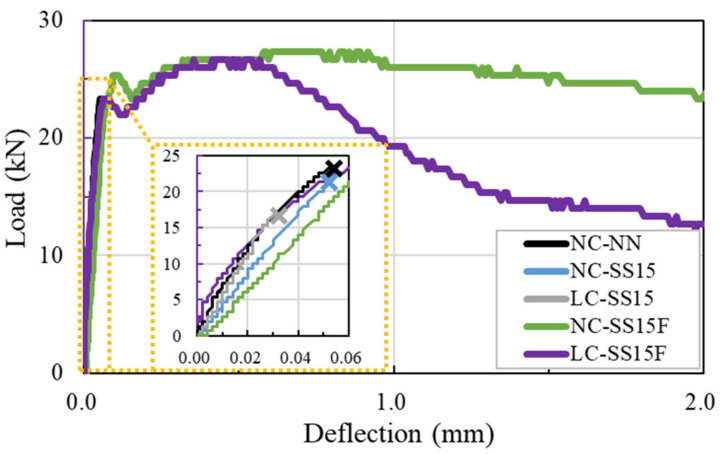
Flexural performance of concrete under four-point flexure test.

Figure 7 shows the flexural behavior of all the concrete specimens. The test specimens without steel fibers (NC-NN, NC-SS15, and LC-SS15) exhibited increased deflection and brittle failure as the load increased. The deflections of NC-NN and NC-SS15 exceeded 0.050 mm, whereas the brittle failure of LC-SS15 occurred when the deflection reached approximately 0.032 mm. Although LC-SS15 had a very low w/b compared to NC-NN and NC-SS15, the flexural performance deteriorated because of the poor load-resistance characteristics of the lightweight coarse aggregate. Unlike in coarse aggregates, cracks penetrate the interior of lightweight coarse aggregates when subjected to external forces [17]. LC-SS15 had a lower deflection and peak load than NC-SS15, owing to these characteristics of lightweight coarse aggregates. The flexural strength of LC-SS15 decreased by approximately 22% compared to that of NC-SS15, and this trend is similar to that in study of Park et al. [48].

Brittle failure did not occur in either NC-SS15F or LC-SS15F, steel-fiber-reinforced concrete (SFRC), even after attaining the peak load. The SFRC showed a constant increase in load up to the first peak load and exhibited hardening behavior after reaching the first peak load. Hardening behavior continued until the peak load was reached, and beyond the peak load, softening and ductile behaviors were observed. As shown in Figure 7, the steel fiber improved the peak load and toughness capacity of the concrete. However, when the lightweight coarse aggregate was added, the effect in the softening section was reduced. On the other hand, ASTM C1609 [41] recommends a method for evaluating concrete toughness based on the energy absorption capacity. In this study, the toughness of the SFRC was calculated for applying the span length of 300 mm. The calculated toughness at L/150 of LC-SS15F was 38.55 J. NC-SS15F had approximately 31% higher toughness than LC-SS15F. Table 4 lists the flexural test values obtained for all the concrete specimens.

### 3.3. Electrical Characterisitcs

#### 3.3.1. Electrical Resistivity

**Figure 8:**
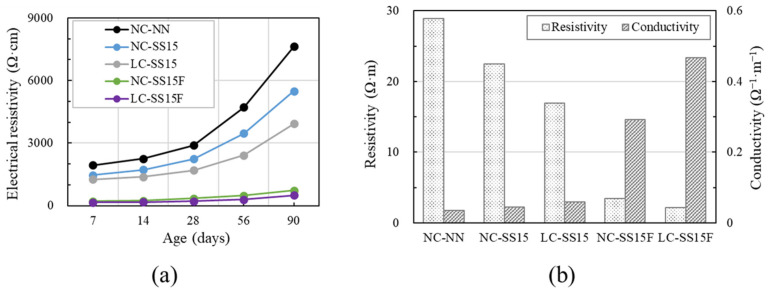
Comparison of electrical properties of concrete: (**a**) electrical resistivity at different ages; (**b**) electrical resistivity and electrical conductivity at 28 days.

Figure 8a shows the results for the electrical properties of the concrete specimens. The electrical resistivity increased with increasing age, and the increment of electrical resistivity also ascended with increasing age. This phenomenon was most evident in NC-NN, probably because the pore water was consumed with age. Ionic and electronic conduction occur in electrical conduction paths, which can cause current flow in concrete. Ion conduction occurs according to the movement of ions (positive ions: Ca^2+^, Mg^2+^, Al^3+^, Fe^2+^, K^+^, and Na^+^; negative ions: OH^−^ and SO_4_^2−^) in the pore water, and electronic conduction occurs according to the movement of electrons in a conductive material [12]. The amount of pore water in concrete decreases, owing to the hydration reaction and drying with increasing age. NC-NN did not contain conductive substances and, therefore, could only be expected to be affected by ion conduction.

Steel slag is a by-product generated during the manufacture of high-strength rebars, and it contains high amount of iron, particularly hematite (Fe_2_O_3_). Fe_2_O_3_ in steel slag can make concrete electrically conductive, and many researchers have reported changes in electrical properties caused by steel slag incorporated into concrete or mortar [34,49,50]. In this study, NC-SS15 exhibited a lower electrical resistivity than NC-NN at all ages. Thus, it appeared that the steel slag was uniformly distributed in the concrete matrix and behaved as a conductive material to form a conductive path, similar to previous studies. Because of these effects, NC-SS15 exhibited a decrease in the electrical resistivity at 28 days by approximately 22.38% compared to that of NC-NN.

Furthermore, the lightweight coarse aggregate appeared to improve the electrical properties of the concrete. The electrical resistivities of LC-SS15 at 7 and 14 days were similar, but the difference in resistivity increased with age. This behavior was attributed to the difference in the chemical component contents between the lightweight coarse aggregate and coarse aggregate. Bottom-ash-based aggregate was used as the lightweight coarse aggregate, and its main components were silica, alumina, and iron [22]. The iron in the lightweight coarse aggregate existed as Fe_2_O_3_. As listed in Table 2, the lightweight coarse aggregate contained approximately 5.1% more Fe_2_O_3_ than the coarse aggregate, and the lightweight coarse aggregate appeared to induce better electrical properties in the concrete than the coarse aggregate. For the electrical resistivity of specimens without steel fibers, the lowest values were derived from LC-SS15 in which 15% of steelmaking slag and lightweight aggregate were substituted at all ages.

However, it is well known that steel fibers improve the electrical conductivity of concrete. When steel fibers were incorporated into concrete, the steel fibers were entangled to form an effective conductive path [13,51]. When 0.75% of the steel fibers were added to the NC-SS15 and LC-SS15 specimens, the decrease in electrical resistivity was significantly reduced. A comparison of the electrical resistivity values at all ages showed that for specimens without fibers, the values ranged between 1200 and 7700 Ω∙cm. In contrast, those of the SFRC specimens ranged between 100 and 800 Ω∙cm. Similar to the relationship between NC-SS15 and LC-SS15 specimens, LC-SS15F exhibited slightly improved electrical properties compared to NC-SS15F. At 90 days, the electrical resistivities of NC-SS15F and LC-SS15F were 725.60 and 503.31 Ω∙cm, respectively. Table 5 represents the electrical resistivity and conductivity of concrete. The conductivities of NC-SS15, LC-SS15, NC-SS15F, and LC-SS15F at 28 days improved by 28.84%, 70.88%, 746.72%, and 1250.96%, respectively, compared to those of NC-NN. Therefore, steel fiber was the most effective material for improving the electrical conductivity of concrete among steel slag, lightweight coarse aggregate, and steel fiber. Furthermore, using lightweight coarse aggregates not only reduced the concrete density but also improved the electrical conductivity of SFRC. Hence, lightweight coarse aggregates and steel fibers should be utilized simultaneously.

#### 3.3.2. Shielding Effectiveness

**Figure 9:**
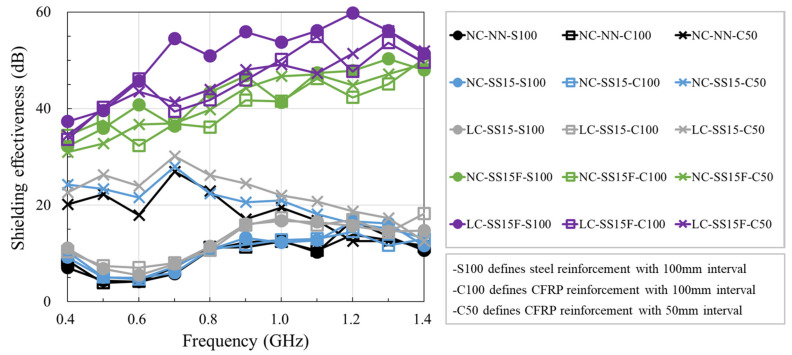
Test results of shielding effectiveness of concrete.

Figure 9 shows the shielding effectiveness for the 0.4–1.4 GHz frequency band. For the S100 series, in which steel rebars were arranged at spacings of 100 mm, the specimens without fibers exhibited similar shielding effectiveness performance. NC-NN, NC-SS15, and LC-SS15 had shielding effectiveness ranging between 4 and 17 dB. The effect of steel slag was confirmed by comparing NC-NN and NC-SS15, and the shielding effectiveness of NC-SS15 improved by approximately 0.9 dB on average compared to that of NC-NN. Based on the measurement results, the steel slag slightly improved the shielding effectiveness, owing to the metallic content in the steel slag, that is, Fe_2_O_3_ and aluminum oxide (Al_2_O_3_) [11]. Wang et al. [11] observed that metallic contents induced the loss of EM waves and increased electrical conductivity because of improved permittivity attributed to steel slag addition. The electrical conductivity of NC-SS15 increased by approximately 29% compared to that of NC-NN. Additionally, Fe_2_O_3_ in the steel slag absorbed the EM waves, and steel slag slightly improved EM shielding [8].

The effect of the lightweight coarse aggregate on the shielding effectiveness was analyzed by comparing NC-SS15 and LC-SS15. Compared to NC-SS15, LC-SS15 exhibited improved shielding effectiveness by approximately 1.7 dB on average, increasing by up to 4.5 dB at 1.0 GHz. The lightweight coarse aggregate slightly improved the shielding effectiveness, similar to the steel slag; this was attributed to the effects of Fe_2_O_3_ and Al_2_O_3_ in the lightweight coarse aggregate. Metallic contents prevent wave propagation [6]. The lightweight coarse aggregate used in this study had an Al_2_O_3_ content similar to that of the coarse aggregate but contained approximately 1.12 times more Fe_2_O_3_. The lightweight coarse aggregate slightly improved the shielding effectiveness of LC-SS15, owing to the difference in the metallic contents. In addition, the pore characteristics of the lightweight coarse aggregate also influenced the shielding effectiveness. The lightweight coarse aggregate had a porous interior, so its EM-wave absorbing characteristics were superior to those of the coarse aggregate. This property tended to improve the shielding effectiveness of LC-SS15 [52].

When steel fibers were incorporated into the concrete, the shielding effectiveness of SFRC increased by at least 23.0 dB for all frequency ranges. NC-SS15F had an increased value by approximately 31.9 dB on average compared to NC-SS15, and LC-SS15F showed an increased value by approximately 38.5 dB on average compared to LC-SS15. As a metallic material, the steel fiber attenuated EM waves by inducing electronic and ionic polarization [53]. NC-SS15F reached 50.3 dB at 1.3 GHz, and LC-SS15F had a value up to 59.8 dB at 1.2 GHz, indicating excellent shielding effectiveness. Hence, steel fiber is an excellent material that can significantly improve the shielding effectiveness and electrical conductivity of concrete. However, the effects of the steel slag, lightweight coarse aggregate, and steel fiber on shielding effectiveness showed a similar trend, regardless of the reinforcement type and spacing.

**Figure 10:**
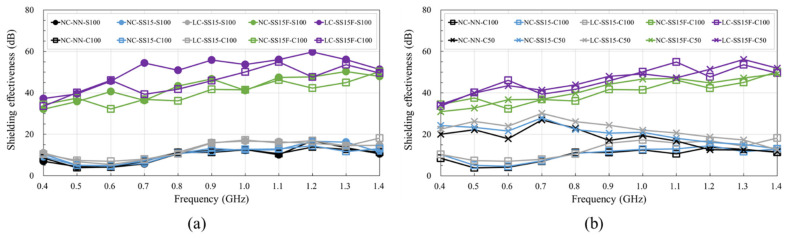
Comparison of shielding effectiveness of concrete for different reinforcement types and spacings: (**a**) type (steel vs. CFRP); (**b**) spacing (100 vs. 50 mm).

Figure 10 shows the variation in shielding effectiveness with frequency for different reinforcement types and spacings. Figure 10a shows a comparison between the S100 and C100 series, with CFRP rebars arranged at 100 mm spacings. The S100 and C100 series showed similar shielding effectiveness performance for all concrete mixes. Overall, fiber-reinforced polymer rebars are non-conductive, but CFRP rebars contain carbon fiber, increasing conductivity; thus, CFRP and steel rebars exhibit similar conductive behavior. Several researchers [8,10,54] found that the conductivity and shielding effectiveness improved with the carbon fiber content. Figure 10b shows a comparison between the C100 and C50 series, in which CFRP rebars were arranged at spacings of 50 mm. The shielding effectiveness of NC-NN, NC-SS15, and LC-SS15 increased by 2.6–3.0 times when the CFRP spacing was reduced from 100 to 50 mm. This phenomenon mainly occurred in the low-frequency band, and at frequencies below 1.0 GHz, the shielding effectiveness increased with decreasing transmission coefficient when reinforcement was arranged [3,5]. The increase in shielding effectiveness with decreasing reinforcement spacing was consistent with the findings reported by Choi et al. [3]. NC-NN, NC-SS15, and LC-SS15 showed the most significant improvement effect with varying CFRP rebar spacing at 0.7 GHz, and the increase values were 19.8, 20.7, and 22.2 dB, respectively. However, for SFRC, no significant effect was observed when the CFRP rebar spacing was reduced. NC-SS15F and LC-SS15F exhibited similar shielding effectiveness with the C100 and C50 series specimens, but the reinforcement did not appear to affect the shielding effectiveness as significantly as the steel fiber did [55]. Therefore, the incorporation of steel fibers is the most effective technique for improving the shielding effectiveness of concrete.

## 4. Conclusions

In this study, the effects of lightweight coarse aggregate and steel fiber on the mechanical and electrical characteristics of concrete were investigated. The main conclusions of this study are as follows:

- The density of concrete decreased when all coarse aggregates were replaced with lightweight coarse aggregates. Despite the addition of steel fibers, LC-SS15F had an equilibrium density of 2119 kg/m^3^, satisfying the density requirement for lightweight aggregate concrete.
- The compressive and flexural strengths decreased when the lightweight coarse aggregate was incorporated. However, the strength and ductile performance improved with the incorporation of steel fibers.
- The lightweight coarse aggregate contained metallic Fe_2_O_3_ and Al_2_O_3_, which improved the electrical conductivity of the concrete. In addition, steel fibers formed the best electrically conductive path by entangling inside the concrete.
- When 0.75% steel fiber content was used, the shielding effectiveness of the concrete increased by at least 23.0 dB. In addition, the CFRP rebars exhibited improved conductivity, and shielding effectiveness similar to those of the steel rebars. However, reducing the CFRP rebar spacing of SFRC from 100 to 50 mm had an insignificant influence on the shielding effectiveness.

## Figures and Tables

**Table 1:** Mix design of concrete specimens.

Mix	w/b *	By Cement Weight Ratio						F (%)	SP ** (%)	AEA ** (%)	Slump (mm)
		W	C	SS	FA	CA	LCA				
NC-NN	0.325	0.32	1.00	-	1.25	1.86	-	-	0.57	0.02	175
NC-SS15	0.325	0.38	1.00	0.18	1.48	2.18	-	-	0.67	0.02	210
LC-SS15	0.200	0.24	1.00	0.18	0.86	-	0.84	-	0.66	0.02	155
NC-SS15F	0.325	0.38	1.00	0.18	1.48	2.18	-	0.75	0.76	0.02	200
LC-SS15F	0.200	0.24	1.00	0.18	0.86	-	0.84	0.75	0.69	0.02	175

w/b: water-to-binder (cement + steel slag) ratio; W: water; C: cement; SS: steel slag; FA: fine aggregate; CA: coarse aggregate; LCA: lightweight coarse aggregate; F: steel fiber; SP: superplasticizer; AEA: air-entraining agent. * w/b was calculated by dividing the weight of water by the weight of the binder. ** Incorporation ratio to binder weight.

**Table 2:** Chemical compositions and physical properties of binders and aggregates.

Content		Binders		Aggregates		
		C	SS	FA	CA	LCA
Chemical composition (% mass)	SiO_2_	17.9	14.2	66.8	61.8	60.9
	CaO	65.5	22.1	1.43	2.51	3.49
	Al_2_O_3_	4.67	11.1	17.6	17.8	17.0
	Fe_2_O_3_	2.79	39.9	2.38	4.54	9.64
	MgO	2.65	3.33	0.85	3.76	1.89
	SO_3_	4.65	0.02	0.25	0.77	0.13
	MnO	0.10	5.59	0.06	0.10	0.11
	TiO_2_	0.27	0.69	0.31	0.76	1.01
	K_2_O	0.95	0.05	4.45	3.72	0.91
	Na_2_O	0.19	0.02	4.32	2.82	1.14
Physical properties	Density (g/cm^3^)	3.15	3.96	2.60	2.67	1.87
	Blaine (cm^2^/g)	3413	4893	-	-	-
	Fineness modulus	-	-	2.88	6.63	6.74

**Table 3:** Physical properties of rebars.

Type	d_b_ (mm)	A_r_ (mm^2^)	f_y_ (MPa)	f_u_ (MPa)	E (GPa)
Steel	10	71.3	400	572	200
CFRP	10	71.3	-	2300	130

**Table 4:** Four-point flexural test results.

Specimen	Max. Load (kN)	Deflection at Max. Load (mm)	Flexural Strength (MPa)	Toughness at L/150 (J)
NC-NN	23.32	0.054	7.0	-
NC-SS15	21.32	0.052	6.4	-
LC-SS15	16.66	0.032	5.0	-
NC-SS15F	27.32	0.578	8.2	50.60
LC-SS15F	26.66	0.354	8.0	38.55

**Table 5:** Electrical resistivity and electrical conductivity of concrete at 28 days.

Specimen	Electrical Resistivity		Electrical Conductivity	
	Value (Ω·m)	S.D.	Value (Ω·m)^−1^	S.D.
NC-NN	28.95	8.30 × 10^−2^	0.03	9.90 × 10^−5^
NC-SS15	22.47	9.43 × 10^−3^	0.04	1.87 × 10^−5^
LC-SS15	16.94	2.07 × 10^−2^	0.06	7.23 × 10^−5^
NC-SS15F	3.42	-	0.29	-
LC-SS15F	2.14	1.89 × 10^−3^	0.47	4.11 × 10^−4^

## Data Availability

The data presented in this study are available on request from corresponding author.
